# Genomic analyses of *Burkholderia* respiratory isolates indicates two evolutionarily distinct *B. anthina* clades

**DOI:** 10.3389/fmicb.2023.1274280

**Published:** 2023-11-21

**Authors:** Amy Pham, James G. Volmer, Daniel C. Chambers, Daniel J. Smith, David W. Reid, Lucy Burr, Timothy J. Wells

**Affiliations:** ^1^Frazer Institute, The University of Queensland, Brisbane, QLD, Australia; ^2^Queensland Lung Transplant Service, The Prince Charles Hospital, Brisbane, QLD, Australia; ^3^Centre for Microbiome Research, Queensland University of Technology, Brisbane, QLD, Australia; ^4^Faculty of Medicine, The University of Queensland, Brisbane, QLD, Australia; ^5^The Adult Cystic Fibrosis Centre and Department of Thoracic Medicine, The Prince Charles Hospital, Brisbane, QLD, Australia; ^6^Australian Infectious Diseases Research Centre, The University of Queensland, Brisbane, QLD, Australia; ^7^QIMR Berghofer Medical Research Institute, Brisbane, QLD, Australia; ^8^Department of Respiratory Medicine, Mater Health, South Brisbane, QLD, Australia; ^9^Mater Research, The University of Queensland, Brisbane, QLD, Australia

**Keywords:** *Burkholderia cepacia* complex, comparative genomics, cystic fibrosis, *Burkholderia anthina*, siderophore

## Abstract

**Introduction:**

The *Burkholderia cepacia* complex (BCC) encompasses a group of at least 22 genetically distinct gram-negatives bacterial species ubiquitous in nature. Recognised as a group of genetically and phenotypically flexible species, the BCC inhabits diverse ecological niches causing both plant and human diseases. Comparative genomic analysis provides an in depth understanding into the population biology, phylogenetic relationship, and genomic architecture of species.

**Methods:**

Here, we genomically characterise *Burkholderia anthina* isolated from patients with chronic lung infections, an understudied pathogen within the *Burkholderia cepacia* complex.

**Results:**

We demonstrate that *B. anthina* is polyphyletic and constitutes two distinct evolutionary lineages. Core- and pan-genome analyses demonstrated substantial metabolic diversity, with *B. anthina* Clade I enriched in genes associated with microbial metabolism in diverse environments, including degradation of aromatic compounds and metabolism of xenobiotics, while *B. anthina* Clade II demonstrated an enhanced capability for siderophore biosynthesis.

**Discussion:**

Based on our phylogenetic and comparative genomic analyses, we suggest stratifying *B. anthina* to recognise a distinct species harbouring increased potential for iron metabolism via siderophore synthesis, for which we propose the name *Burkholderia anthinoferum* (sp. nov.).

## Introduction

The *Burkholderia cepacia* complex (BCC) represents a group of metabolically versatile and highly adaptable bacteria found inhabiting diverse ecological niches. In the early 1980’s, the BCC emerged as an opportunistic pathogen in patients with cystic fibrosis (*CF*), gaining notoriety due to its intrinsic resistance to antibiotics and propensity to spread rapidly within the community causing devasting outbreaks (Speert et al., 2002; Vandamme et al., 2003). Treatment and management of *Burkholderia* infections still represents a long-standing challenge for clinicians, with infection considered a contraindication for lung transplantation due to the significant post-operative mortality associated with the bacteria (Aris et al., 2001; Hauser et al., 2011; Ramos et al., 2016). Infections by the BCC pose a serious therapeutic challenge, and hence, understanding their genomic architecture and metabolic potential is critical to understand their pathogenesis. Encompassing a group of at least 22 genetically distinct species, the BCC features some of the largest and most complex bacterial genomes described to date, characterised by a multireplicon structure ranging from 6 to 9 Mb. Harbouring bacteriophages, several genomic islands, and an extensive repository of insertion sequence elements, the large genome size of the species underpins their genetic and metabolic versatility (Baldwin et al., 2004; Holden et al., 2009).

The taxonomy of *Burkholderia* has undergone extensive reclassification over the last few decades and continues to expand as new lineages and species are proposed (Vandamme et al., 1997; Coenye et al., 2001a,b; Vandamme et al., 2002; Mullins and Mahenthiralingam, 2021). Historically, the BCC was thought to constitute a single species known as *Pseudomonas cepacia*, however in 1992 was renamed to recognise several distinct genomovars. Following the implementation of *recA* sequence analysis, taxonomic distinction of species within the BCC expanded with new species being rapidly described. The genetic diversity within species was also recognised, with the taxonomy of *B. cenocepacia* extended to include four distinct subgroups within the bacterial population known as lineage III-A, III-B, III-C and III-D (Mahenthiralingam et al., 2000; Vandamme et al., 2003). Likewise, *B. cepacia* was also divided into separate sub lineages following the identification of types AD, AW, and K, with group K now consisting of two validly named species, *B. contaminans* and *B. lata* (Vermis et al., 2002; Vanlaere et al., 2009; Depoorter et al., 2020). Although the *recA* based identification scheme has been widely implemented in epidemiological investigations its utility to resolve phylogenetic relationships among other closely related species is limited (LiPuma et al., 1999). The advent of sequencing technologies has provided a better means to genomically characterise species and evaluate phylogenetic relationships. Indeed, recent comparative genomic analyses revealed novel genomic taxa within the BCC, with the proposal of new species *B. paludism*, *B. reimsis*, and *B. orbicola* (Ong et al., 2016; Esmaeel et al., 2018; Wallner et al., 2019; Morales-Ruiz et al., 2022).

*B. anthina* represents an understudied pathogen within the BCC. From the 2,425 sequenced BCC genomes deposited in the National Center for Biotechnology Information (NCBI) database to date, only 31 (1.27%) correspond to *B. anthina* strains, with most isolates originating from an environmental source. In the current study, we examined phylogenetic relationships among *Burkholderia* species within the BCC and conducted comparative genomic analyses of *B. anthina*. Whole genome sequencing of *Burkholderia* isolates collected from patients with *CF* in Queensland, Australia identified a further five strains of *B. anthina.* Genomic characterisation of *B. anthina* genomes identified two evolutionarily distinct clades with considerable genomic divergence. Based on phylogenetic evidence, whole genome average nucleotide identity, and comparative genome analyses, we propose the division of *B. anthina* into two separate species: *B. anthina* and *B. anthinoferrum* (sp. nov.).

## Methods

### Isolation of *Burkholderia* spp. from sputum and culture conditions

Cases of *Burkholderia* infections detected in patients with *CF* attending The Prince Charles Hospital and the Mater Hospital, Queensland, Australia, between 2017 and 2022 were included in the study. Clinical specimens were collected from the Prince Charles Hospital as per the Research Collaboration Agreement HREC/17/QPCH/277, and the Mater Hospital as per HREC/14/QPAH/275. Bacterial isolation was performed from expectorated sputum samples. Briefly, a direct inoculum from sputum was plated out onto *Burkholderia cepacia* selective agar (BCSA) and incubated at 37°C under aerobic conditions for 48 h. The medium, supplemented with crystal violet, vancomycin and gentamicin, inhibits growth of gram-positive bacteria and gram-negative bacilli *Pseudomonas*, for increased selectivity of *Burkholderia*. Bacteria were grown under aerobic conditions in lysogeny broth (LB) (1% (w/v) tryptone, 1% (w/v) yeast extract, 0.5% (w/v) sodium chloride) at 37°C, with shaking at 220 rpm overnight.

### Whole genome sequencing and assembly of *Burkholderia* spp. isolate genomes

For genomic DNA extraction, bacterial cultures were grown in 5 mL of LB, followed by overnight incubation at 37°C with shaking at 220 rpm. Genomic DNA was extracted using a QIAamp DNA Mini Kit (Qiagen) as per the manufacturer’s instructions. DNA concentration and purity were assessed using a Nanodrop 1,000 spectrophotometer. Whole genome sequencing (WGS) was performed by MicrobesNG^1^ using the Illumina sequencing platform with 2 × 250 bp pair-end reads. Genome assembly was conducted by MicrobesNG using their standard analysis pipeline. Briefly, Kraken was used to identify the closet available reference genome (Wood and Salzberg, 2014). The reads were mapped to the reference genome using BWA mem to assess the quality of the data (Li and Durbin, 2009). *De novo* assembly of the reads was conducted using SPAdes (Bankevich et al., 2012). Genome annotation was completed using Prokka (Seemann, 2014). Reference genomes were downloaded from the NCBI database^2^ (Supplementary Table 1).

### Quality assessment, phylogeny, and average nucleotide analysis of *Burkholderia* spp. isolate genomes

Genome quality assessment and assembly metrics were assessed using CheckM (v1.1.2) (Parks et al., 2015). Genomes with an estimated completeness of ≥90% and contamination of ≤5% were determined high-quality and included for comparative analysis. Multi-locus sequence typing (MLST) was determined by querying genomes against the *Burkholderia cepacia* complex typing database^3^ available on PubMLST (Jolley et al., 2018). Under the MLST scheme seven independent loci comprising of genes *atpD*, *gltB*, *gyrB*, *recA*, *lepA*, *phaC*, and *trpB* are assessed to identify differences in the allelic profile to define the sequence type (ST). Taxonomic classification was assigned using the Genome Taxonomy Database Toolkit (GTDB-Tk; v2.2.4) with reference database r207. A concatenated set of 975 core genes were produced using BPGA and the resultant output used to predict phylogeny (Parks et al., 2018; Supplementary Data 1). Phylogenetic analysis was conducted using RAxML (v8.2.13) (Stamatakis, 2014) and visualised in iToL (v6.8.1).^4^ Average nucleotide identity (ANI) was determined using FastANI (v1.1) and visualised using the ‘pheatmap’ (v1.0.12) and ‘ggplot’ (v3.1.3) packages in Rstudio (v4.1.3) and R (v4.2.3). Genomes were annotated through EnrichM (v0.4.15^5^) using the ‘annotate’ function, with the input parameter ‘--orthologs’ to annotate orthologous genes.

### Comparative genomic analysis of *Burkholderia anthina* genomes

A total number of 34 *B. anthina* genomes were included in the comparative genomic analyses. Statistical analyses for comparing distribution of genome size, number of CDSs, and coding density were determined using an unpaired *t*-test. The Bacterial Pan Genome Analysis (BPGA) pipeline using the USEARCH tool was used to cluster protein sequences into gene families (Chaudhari et al., 2016). A sequence identity cut-off value of 80% was used for orthologous clustering. The pan-genome profile analysis tool was used to determine the pan and core genome size, calculated with 100 iterations. Data are calculated as a function of genomes introduced into the analysis. The pan-genome functional analysis tool was used to assign KEGG Ortholog pathways to protein sequences, and execute comparative functional analysis for core, accessory, and unique genes. Principle component analysis (PCA) of KEGG orthologs was performed using the ‘factoextra’ (v1.0.7), ‘ggplot2’ (v3.4.4), ‘purrr’ (v1.0.2), ‘scatterplot3d’ (v0.3-44), ‘ade4’ (v1.7-22), and ‘gdata’ (v3.0.0) packages in Rstudio (v4.1.3) and R (v4.2.3). Comparative genomics based on KEGG Orthology was completed using EnrichM (v0.4.15; see footnote 5). Genomes were annotated using the ‘annotate’ function, with the input parameter ‘--ko’ used to annotate KEGG Orthology. Statistical analyses between clades were performed in EnrichM by Fisher’s exact test. Corrected *p*-values of <0.05 were considered significant. Secondary metabolite gene clusters were predicted using anti-SMASH (v7.0.0^6^) using the parameter ‘relaxed’ to identify hits (Blin et al., 2023). *In silico* characterisation of the ethylenediaminesuccinic acid hydroxyarginine (EDHA) biosynthetic gene cluster was conducted through BLASTn searches. *Streptomyces* sp. MA5143a was used as the reference sequence and encodes for an EDHA operon containing four genes denoted *AesA, AesB, AesC,* and *AesD.* The genetic organisation of the EDHA gene cluster was visualised using the ‘gggenes’ (v0.5.1) and ‘ggplot2’ (v3.4.4) packages in Rstudio (v4.1.3) and R (v4.2.3). Identification of iron genes and iron gene operons was predicted using the bioinformatic tool FeGenie (Garber et al., 2020). The RAST server (v2.0) was used to annotate genomes and identify genes associated with iron metabolism, specifically siderophore biosynthesis (Aziz et al., 2008; Overbeek et al., 2014; Brettin et al., 2015).

## Results

### Characterisation of *Burkholderia* isolated from patients with *CF* in South-East Queensland identifies clonal strains shared among patients.

Taxonomic analysis of *Burkholderia* previously relied on *recA* gene sequence analysis, with the molecular approach proving to be a valuable tool in the early distinction between species. Although widely implemented and used in epidemiological investigations, its utility to resolve in depth phylogenetic relationships among closely related species within the BCC is limited. To further understand the evolutionary relationships of *Burkholderia*, we performed genomic analyses among and within species of the BCC. Here, we genome sequenced 18 clinical isolates cultured from patients with *CF* in Queensland, Australia. The quality of each genome assembly was assessed using CheckM, with genomes more than 95% complete, and < 5% contaminated retained for analysis (Table 1). A total of 177 *Burkholderia* genomes were included as part of this study, including 159 publicly available genomes, with *B. gladioli* selected as the outgroup species. The genome size of *Burkholderia* strains isolated in this study ranged from 6.3 to 8.3 Mbp, with a G + C content of 67%, and predicted number of coding genes ranging from 5,600–7,600 genes (Table 1).

**Table 1:** Genome statistics of *Burkholderia* isolates included in this study.

Isolate ID	Contamination (%)	Completeness (%)	Coverage	Genome size (Mb)	GC content (%)	Contigs	N50 contigs (bp)	Predicted genes	GTDB Classification
BCCIQ01A	0.63	99.87	91.2	7.66	67	137	149,793	6,904	s__*Burkholderia cenocepacia*
BCCIQ02A	0	99.19	32.8	6.43	67	160	82,737	5,796	s__*Burkholderia multivorans*
BCCIQ03A	0.2	99.19	37.5	6.42	67	172	77,986	5,792	s__*Burkholderia multivorans*
BCCIQ04A	0.74	100	181.5	7.54	67	82	423,811	6,784	s__*Burkholderia anthina*_A
BCCIQ04B	0.54	100	53.5	7.53	67	116	208,655	6,809	s__*Burkholderia anthina*_A
BCCIQ04C	0	99.58	33.4	6.43	67	112	159,148	5,768	s__*Burkholderia multivorans*
BCCIQ04D	0.2	99.6	76.9	6.43	67	128	126,777	5,768	s__*Burkholderia multivorans*
BCCIQ05A	1.57	99.85	32.5	8.27	67	345	67,270	7,641	s__*Burkholderia cenocepacia*
BCCIQ06A	1.76	100	39.7	7.63	68	278	63,476	6,723	s__*Burkholderia gladioli*
BCCIQ07A	0.54	100	262.7	7.54	67	213	94,639	6,843	s__*Burkholderia anthina*_A
BCCIQ07B	0	99.6	42.5	6.44	67	68	590,938	5,769	s__*Burkholderia multivorans*
BCCIQ07C	0.34	100	30.5	7.53	67	218	79,729	6,839	s__*Burkholderia anthina*_A
BCCIQ07D	0.58	100	34.5	7.47	67	198	97,875	6,714	s__*Burkholderia anthina*_A
BCCIQ07E	0	98.96	54.5	7.30	67	124	178,011	6,526	s__*Burkholderia cenocepacia*_B
BCCIQ08A	0	99.6	62.9	6.37	67	86	317,180	5,617	s__*Burkholderia multivorans*
BCCIQ08B	0	99.6	188.7	6.36	67	70	381,777	5,613	s__*Burkholderia multivorans*
BCCIQ09A	0.38	99.35	211.6	7.38	67	145	176,679	6,718	s__*Burkholderia cenocepacia*
BCCIQ09B	0.2	99.35	172.5	7.04	67	139	180,290	6,370	s__*Burkholderia cenocepacia*

Taxonomic classification was determined using GTDB-Tk (v2.2.4) with reference database r207. Genome quality and assembly metrics were assessed using CheckM (v1.1.2).

**Figure 1:**
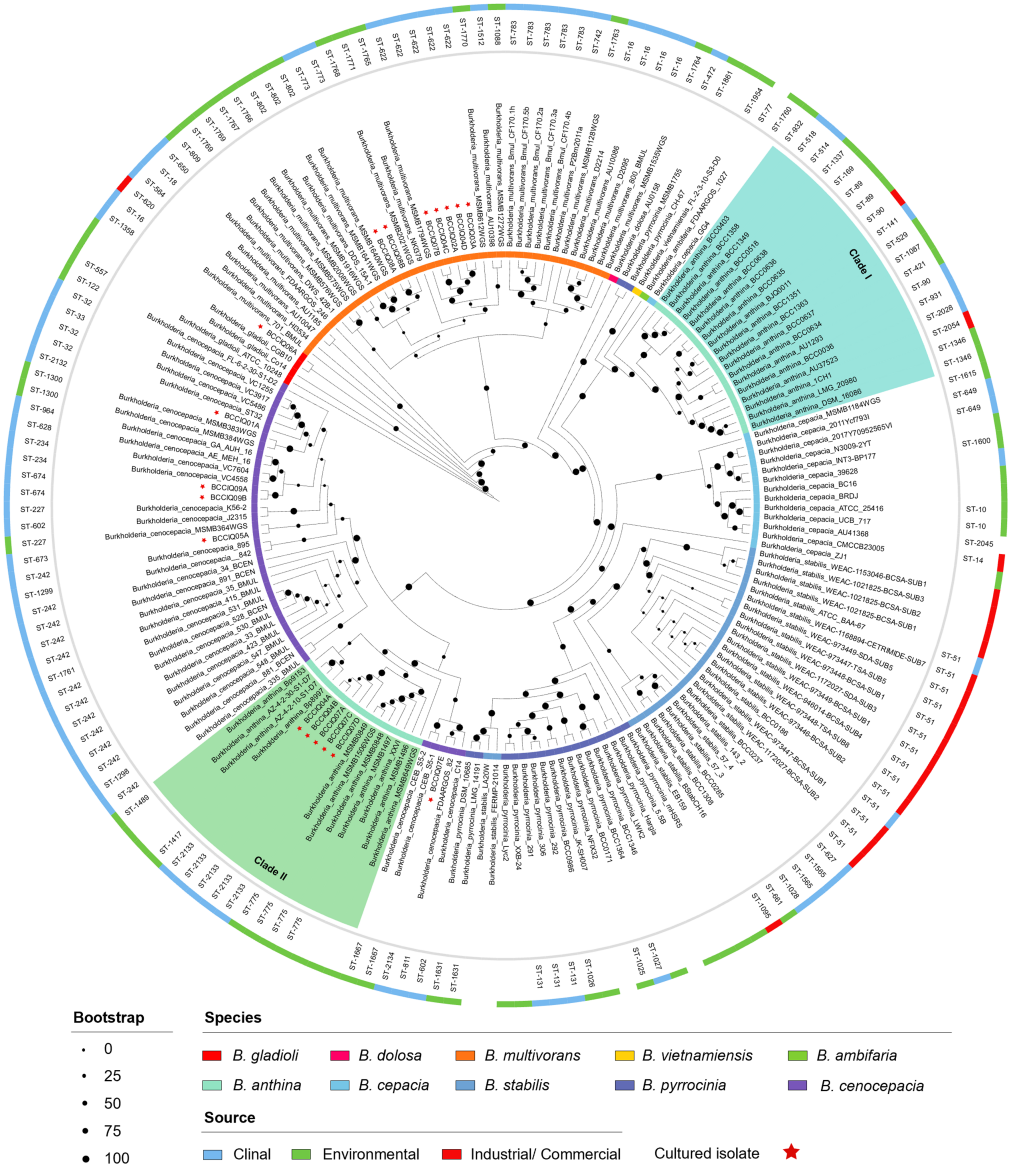
Phylogenetic distribution of *Burkholderia* spp. genomes. Concatenated core gene files were produced using BPGA, with *B. gladioli* used as the outgroup. Phylogeny was inferred using RAxML (v8.2.13) and visualised by iToL (v6.8.1). Cultured isolates recovered from this study are denoted BCCIQ, boldfaced in red, and identified by a red star. Phylogenetic placement of *B. anthina* (shaded green) indicates two distinct evolutionary clades within the species group.

Phylogenetic analysis was performed using a set of 975 core genes produced using BPGA and inferred using RAxML. MLST analysis was performed to inform strain relatedness among isolates. As part of this study, we obtained four isolates belonging to *B. cenocepacia* III-A, and one isolate belonging to *B. cenocepacia* III-B. All *B. cenocepacia* isolates cultured in this study clustered separate from each other and displayed a unique ST (Figure 1). While five of the seven cultured *B. multivorans* isolates (BCCIQ02A, BCCIQ03A, BCCIQ04C/D and BCCIQ07B) displayed the same ST-622 and clustered tightly together. Phylogenetic placement of *Burkholderia* genomes identified several distinct phylogenetic clades within *B. anthina.* Notably, all five *B. anthina* isolates clustered within Clade II, displayed the same ST-2133, and represent the first clinical isolates reported within the group. Based on a well-supported phylogeny, it is evident that *B. anthina* Clade II represents a truly distinct group to *B. anthina* Clade I. As shown in Figure 1, *B. anthina* is polyphyletic with Clade I, as well as *B. vietnamiensis,* and *B. ambifaria,* diverging earlier than *B. anthina* Clade II. Phylogenetic placement of *B. anthina* Clade II suggest that this lineage shares a more recent common ancestor with *B. cenocepacia*, *B. cepacia, B. stabilis,* and *B. pyrrocinia.*

### Comparative genomic analyses reveal hidden genetic diversity of *B. anthina*

**Figure 2:**
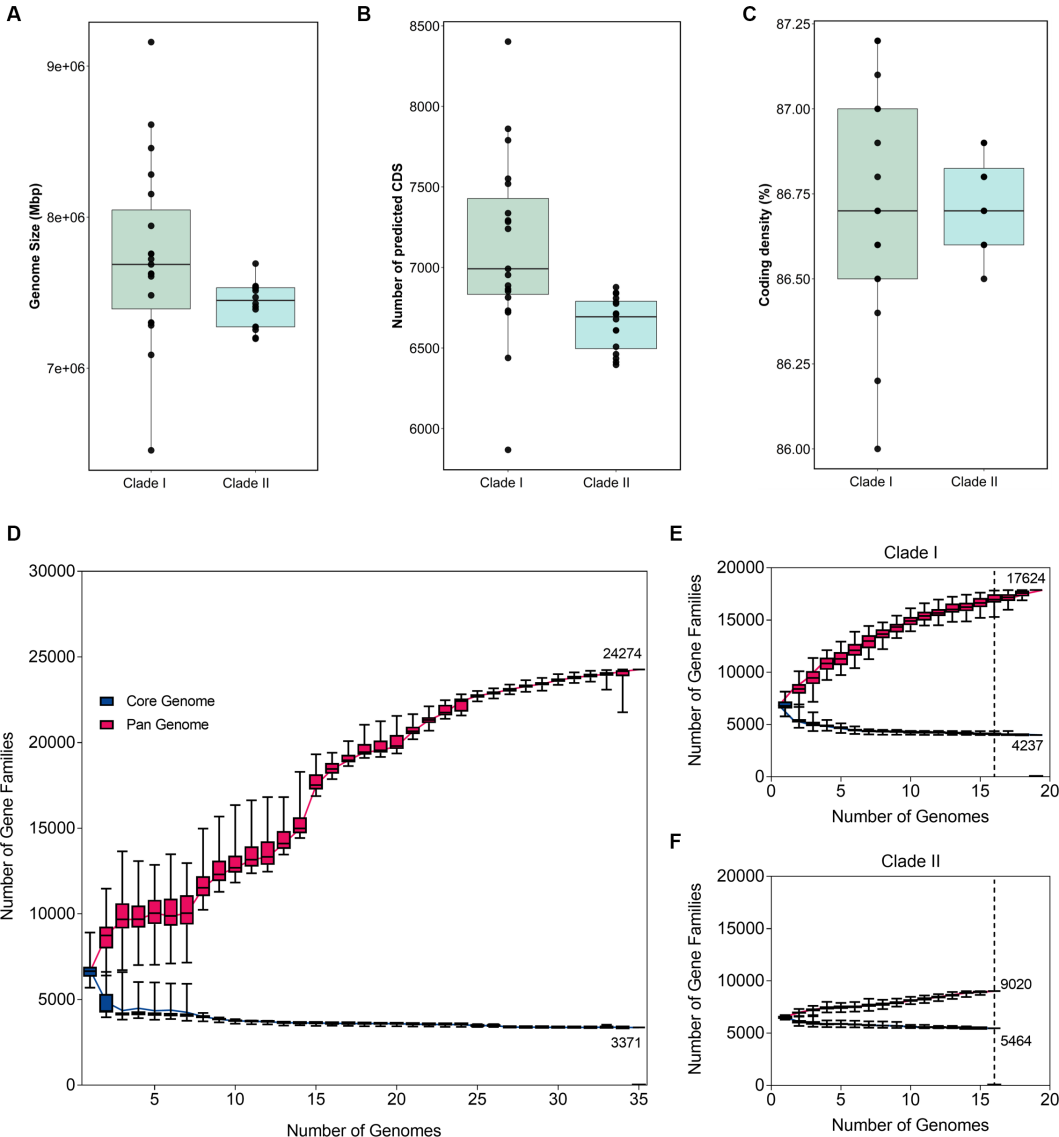
Variations in genomic architecture between *B. anthina* genomes. Comparison of **(A)** genome size, **(B)** coding density, and **(C)** predicted coding sequences in *B. anthina* Clade I (19 genomes) compared with *B. anthina* Clade II (16 genomes). Genomic statistics were assessed using CheckM (v1.1.2) and the BPGA analysis tool. Comparative analysis of the pan and core genome of **(D)** all *B. anthina* genomes, **(E)** *B. anthina* Clade I, and **(F)** *B. anthina* Clade II. The pan-genome profile was determined using the BPGA software, calculated with 100 iterations. The number of gene families is graphed as a function of the number of genomes sequentially added. The dotted line denotes the reference point at which the pan-core genome profile can be accurately compared across the clades. At 16 genomes, the pan-core plot of Clade I showed 17,624 genes in the pan-genome, compared to Clade II which contained 9,020 genes.

The phylogenetic separation in *B. anthina* is further reflected in the genomic architecture between the two clades. *B. anthina* Clade I genomes have a significantly larger genome than *B. anthina* Clade II (*p* < 0.0001), with a mean size of 7.7 Mbp as compared to 7.4 Mbp (Figure 2A). Accordingly, *B. anthina* Clade I have a significantly greater number of predicted coding sequences than *B. anthina* Clade II (*p* = 0.0029), with an average of 472 more coding sequences (Figure 2B). The coding density of both clades was comparable with an average of 87% (Figure 2C). The pan-genome of *B. anthina* demonstrated characteristics of an “open” pan genome, composed of a core genome of 3,371 orthologous genes (12.2%) shared by all strains, and a pan genome of 24,274 genes (87.8%) (Figure 2D). The gene accumulation rarefaction curve increased with the addition of each new genome, reflecting an increase in the number of gene families, and expanding the gene pool diversity. When analysed within their respective clades, Clade I (Figure 2E) and Clade II (Figure 2F) genomes also revealed an “open” profile. At an analysis of 16 genomes, Clade I demonstrated the greatest diversity among strains with only 4,237 core genes (19.4%) and 17,624 (80.6%) pan genes. This contrasts with Clade II which had 5,464 core genes (38%) and 9,020 (62%) pan genes. The pan and core genome profile of *B. anthina* Clade I highlight the genetic variability within the group and reflects the differences among genomes.

### Phylogenomic analysis indicates two evolutionary distinct clades within *B. anthina*

**Figure 3:**
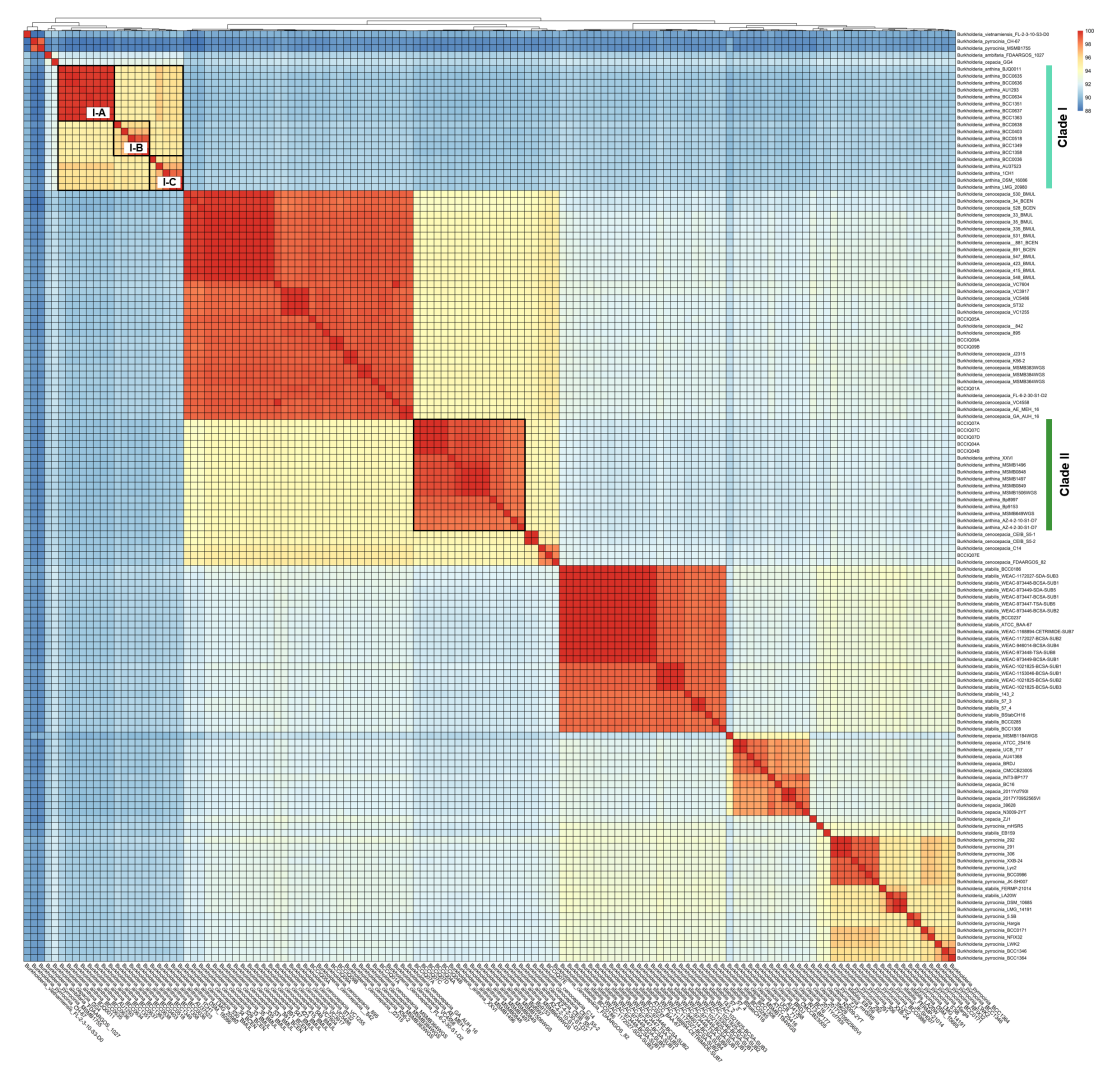
Pairwise average nucleotide identity comparison between *B. anthina* genomes and closely related *Burkholderia* species. Pairwise ANI was calculated using FastANI (v1.1) and visualised using the ‘pheatmap’ (v1.0.12) and ‘ggplot’ (v3.1.3) packages in Rstudio (v4.1.3) and R (v4.2.3). The heatmap depicts phylogenetic relationships, with ANI values clustered according to their distance patterns. ANI score is calculated from BLAST hits between orthologous genes of the core genome.

Whole genome average nucleotide identity (ANI) was calculated to determine strain relatedness and assess the species boundaries of *B. anthina* and closely related species as indicated by phylogenetic placement. A threshold of 98% ANI is considered the cut-off for strain classification within species, whereas a threshold of 95% ANI is considered the cut-off for species delineation (Goris et al., 2007; Jain et al., 2018). Consistent with the relationships inferred by phylogenetic analyses, ANI evaluation confirmed the identification of two distinct lineages within *B. anthina* (Figure 3). Pairwise ANI of *B. anthina* Clade I and Clade II demonstrated 90% pairwise identity, indicating that the two groups represent distinct species according to the operational 95% ANI threshold commonly used for species designation and appropriate for *Burkholderia*. Genomes clustering with *B. anthina* Clade I appeared to be genetically variable with further clusters designated I-A, I-B, and I-C identified within the group. Among these clusters, genomes displayed a pairwise ANI between 94.7–95.8%, bordering the threshold for species demarcation. In comparison *B. anthina* Clade II harboured highly similar strains with a pairwise ANI ≥ 98%. Assessment of species boundaries between *B. anthina* Clade II and *B. cenocepacia* confirmed that these groups are distinct to each other, with less than 95% pairwise identity (Supplementary Table 2). Taken together, these analyses corroborate our findings that there are two distinct lineages within *B. anthina*.

### Analysis of secondary metabolites reveals diverse metabolic potential

**Figure 4:**
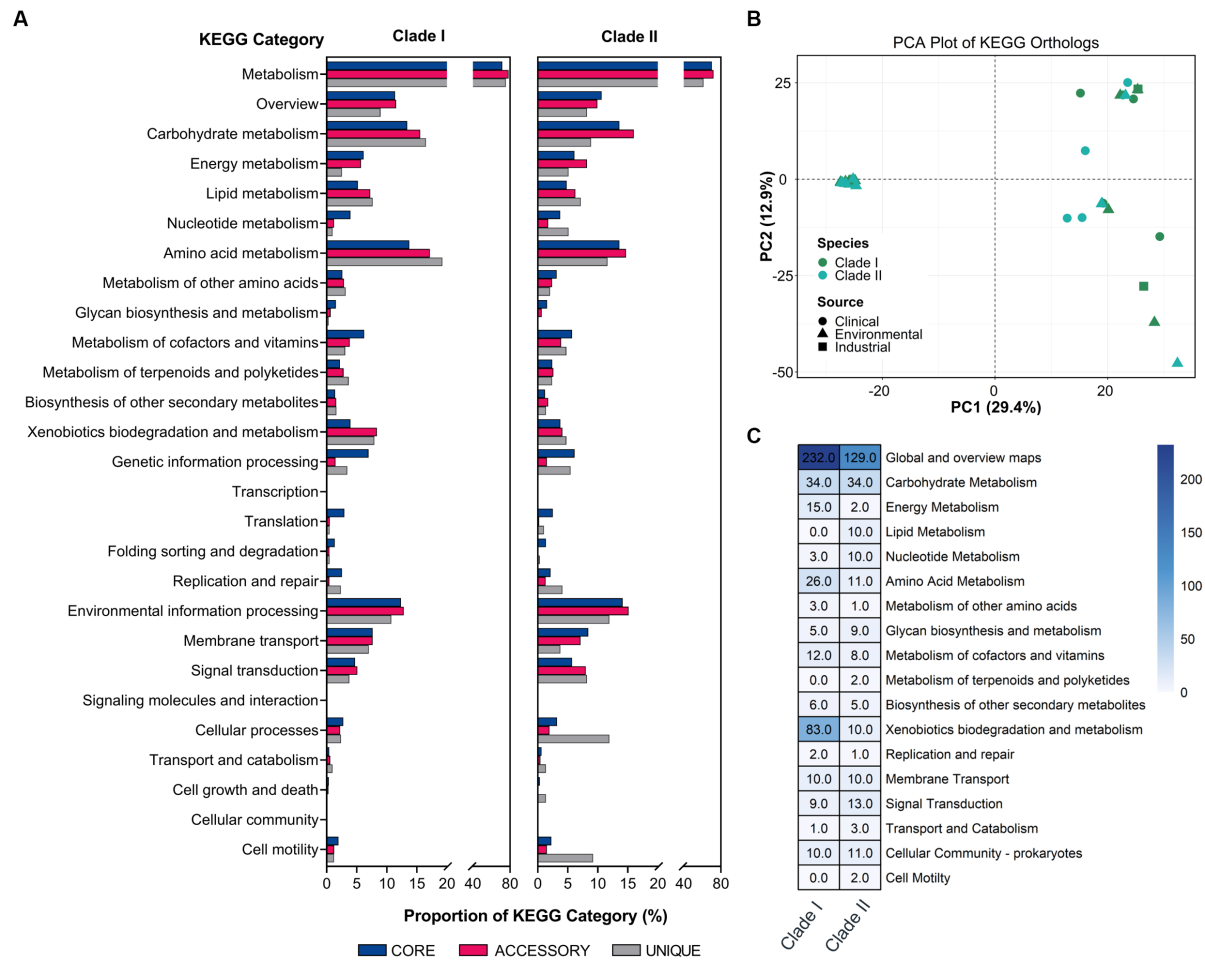
Functional analysis of major KEGG categories in *B. anthina*. **(A)** Comparison of KEGG distribution profile of core, accessory, and unique gene sets in Clade I and Clade II represented as a percentage. **(B)** Principal component analysis of KEGG orthologs between clades. Gene annotations were generated using EnrichM (v0.4.15; https://github.com/geronimp/enrichM) using the ‘—KEGG’ analyses. Genomes are coloured according to species classification, with the shape of the symbol referring to the source of the isolate, as indicated by the legend. **(C)** Differential enrichment of KEGG pathways in *Burkholderia anthina* displayed. KO annotation and statistical analyses were performed using the ‘annotate’ and ‘enrichment’ functions of EnrichM (v0.4.15; https://github.com/geronimp/enrichM). *B. anthina* genomes were grouped by clades and compared by Fisher’s exact test, where KOs with corrected *p* values of <0.05 were retained and considered significant.

To explore the functional differences of the different *B. anthina* clades, the pangenome was analysed according to their KEGG ortholog pathways. Both groups demonstrated a similar KEGG ortholog distribution profile regarding the relative proportion of core, accessory, and unique genes assigned to each KEGG pathway. However, *B. anthina* Clade II, displayed a higher relative proportion of unique genes associated with cellular processes and motility. KEGG categories associated with metabolism, including amino acid and carbohydrate metabolism were the most abundant categories across both *B. anthina* clades for core, accessory, and unique genes (Figure 4A). Interestingly, *B. anthina* genomes displayed substantial variance in KEGG orthologs between and within the identified clade groups (Figure 4B). To highlight clade specific adaptations and identify distinct biological interactions, we performed a KEGG ortholog enrichment analysis using a Fisher’s exact test. An examination of enriched KEGG orthologs revealed 722 genes differentially enriched among the two groups, with 451 (62.5%) genes enriched in Clade I and 271 (37.5%) genes enriched in Clade II (Figure 4B; Supplementary Table 3). KEGG orthologs associated with metabolism represented the most prominent KEGG category enriched in both groups. KEGG categories associated with genetic information processing, environmental information processing, and cellular processes were also enriched, albeit at a lower proportion. Genes associated with global overview and map, carbohydrate metabolism, amino acid metabolism, and xenobiotics biodegradation and metabolism showed frequent enrichment in both groups. In terms of genes encoding functions for metabolism, Clade I showed greater functional enrichments linked to metabolic pathways, microbial metabolism in diverse environments, and degradation of aromatic compounds. Of ecological importance, xenobiotics biodegradation and metabolism were largely enriched in Clade I, with gene functions specific to benzoate degradation, xylene degradation, chlorocyclohexane and chlorobenzene degradation, dioxin degradation, toluene degradation, steroid degradation, and styrene degradation.

**Figure 5:**
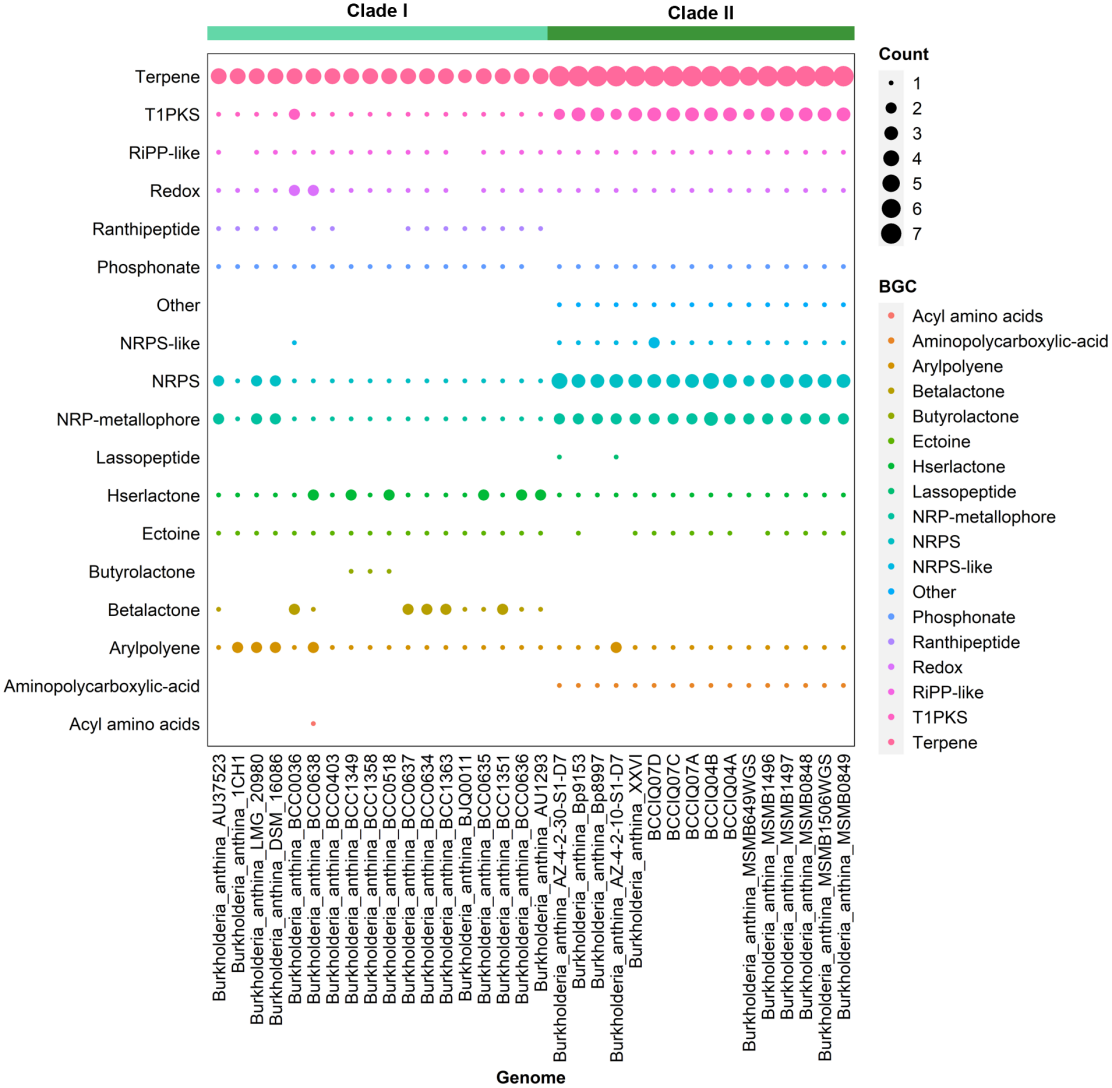
Presence of biosynthetic gene clusters in *B. anthina* genomes. BGCs were detected using Anti-SMASH (v7.0.0). The size of the dot represents BGC count. Genomes are clustered according to clade designation.

**Figure 6:**
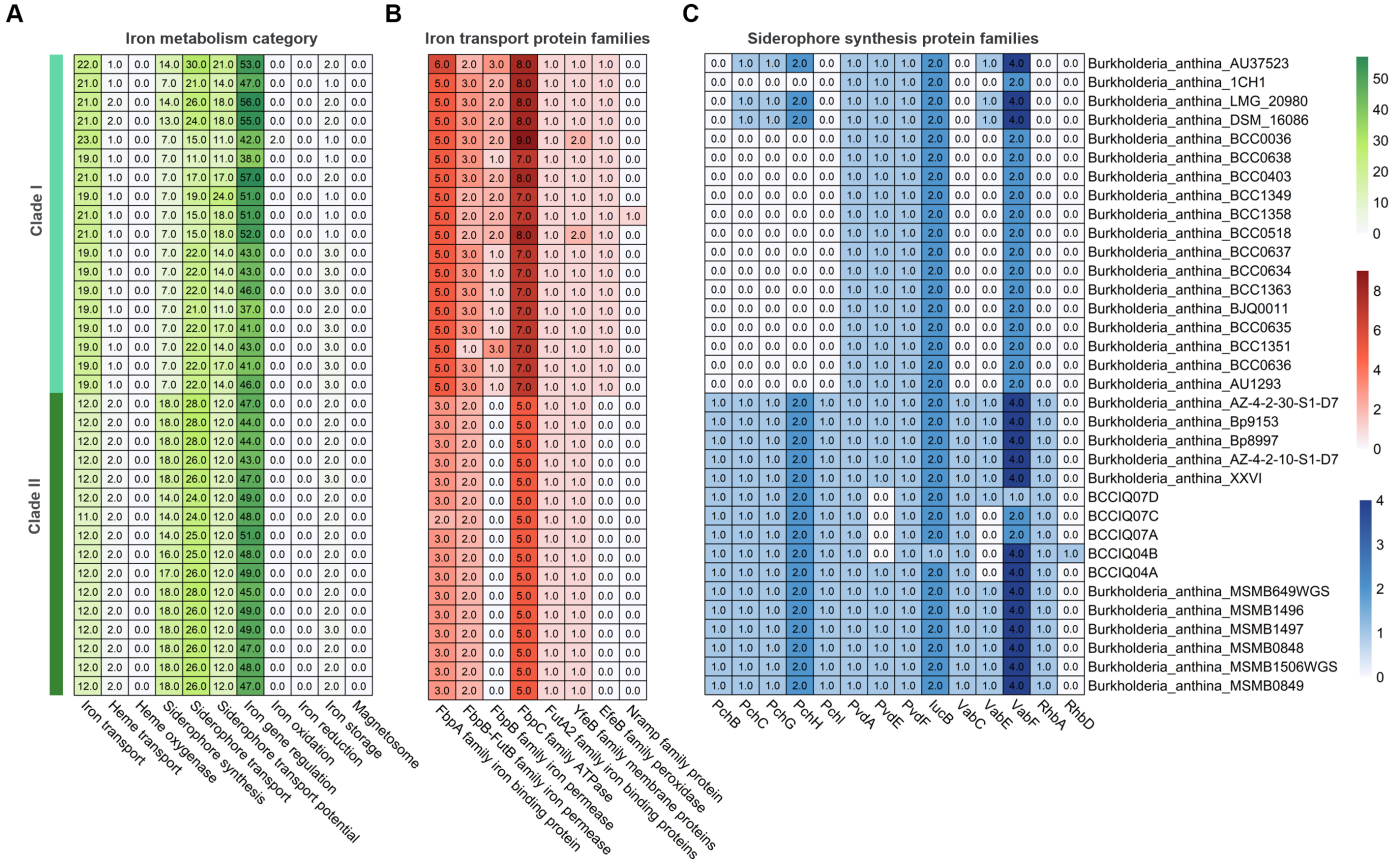
Heatmap showing gene count of different iron metabolism categories in *B. anthina* genomes. **(A)** Gene count involved in iron transport, iron oxidation and reduction, iron storage, and iron regulation were predicted using FeGenie. Protein families associated with **(B)** iron transport and **(C)** siderophore synthesis.

Analysis of secondary metabolites identified 18 different biosynthetic gene clusters (BGC) in *B. anthina* with clear differences between Clades I and II (Figure 5). Genes clusters inferred to encode the biosynthetic pathway for terpene production (186), non-ribosomal peptide synthases (NRPS) (72), type I polyketide synthase (T1PKs) (65), and NRP-metallophone (56) were most abundant. BGC counts associated with terpene production, NRPS, and T1PKs were higher in Clade II than that of Clade I. While Clade I demonstrated a broader distribution of specialised metabolite cluster types, with ranthipeptide, betalactone, and butyrolactone exclusively present in Clade I, albeit not detected across all genomes. Aminopolycarboxylic-acid and other were exclusively present in all genomes within Clade II. The aminopolycarboxylic-acid gene cluster is predicted to encode the biosynthesis of ethylenediaminesuccinic acid hydroxyarginine (EDHA), an iron responsive regulated zwitterionic siderophore. To validate the presence of the predicted EDHA gene cluster we bioinformatically identified the operon within the genome, using *Streptomyces* sp. MA5143a as the reference sequence. As predicted, all Clade II *B. anthina* genomes harboured homologues for all four genes, allowing for the biosynthesis of EDHA a newly described siderophore (Supplementary Figure 1). To gain insight into the overall iron metabolism potential of *B. anthina*, the bioinformatic tool FeGenie was used identify genes related to iron acquisition, storage, and redox cycling. Genes associated with iron gene regulation and iron storage were comparable across the two clades, and gene families related with heme oxygenase, iron reduction, or magnetosome formation were absent across all genomes (Figure 6A). Generally, a greater number of gene families associated with iron transport were identified in Clade I (Figure 6B), while gene families associated with siderophore synthesis were higher in *B. anthina* Clade II (Figure 6C). Genome annotations by RAST predicted on average 31 more genes associated with siderophore synthesis in Clade II compared to Clade I, highlighting the extended siderophore assembly capabilities of the group (Supplementary Table 4). All *B. anthina* genomes encoded genes required for ornibactin synthesis, while only Clade II genomes encoded genes for pyochelin synthesis, and at least 20 genes putatively involved in siderophore biosynthesis. Interestingly, four phylogenetically closely related strains (*B. anthina* 1CH1, *B. anthina* AU37523, *B. anthina* DSM 16086, and *B. anthina* LMG 20980) identified in Clade I harboured the genes *FhuB, FhuC,* and *FhuD* required for uptake of ferric aerobactin. The synthesis and transport of the aerobactin siderophore is frequently produced by several *Enterobacteriaceae*, including *Escherichia, Vibrio, Salmonella,* and *Shigella* (Waters and Crosa, 1988; Sheldon et al., 2016). Given that aerobactin biosynthesis is not an established feature of *Burkholderia* it is likely these genes were horizontally acquired. Overall, these results highlight clear genomic and metabolic differences between the two clades identified in *B. anthina* and provides strong evidence that they should be considered two different species.

## Discussion

The *Burkholderia cepacia* complex represents a significant group of organisms heterogeneously distributed in the natural environment, and contains species of ecological, pathogenic, and biotechnological interest (Coenye and Vandamme, 2003; de Los Santos-Villalobos et al., 2018). Harbouring species that are metabolically versatile and highly adaptable, the BCC can inhabit diverse ecological niches, and tolerate environmental conditions subject to nutrient limitation, toxic compounds, antibiotics, and antimicrobial peptides. The genomic and phenotypic flexibility of these microbes is in part explained by the large coding capacity of these genomes. The advent of next-generation sequencing technologies has facilitated the advancement of comparative genomics to elucidate the genomic architecture, population biology, and phylogenetic relationships among species (De Smet et al., 2015). Through genomic analysis of the BCC, we have identified a distinct clade within *B. anthina* which ancestrally diverged from its sister clade. Comparative analysis uncovered remarkable genetic and metabolic diversity between the two clades. Based on our phylogenetic analyses, ANI, and comparative genomic studies, we suggest amending *B. anthina* to recognise a new species. We propose that the name *B. anthina* is retained to represent all strains clustering with the type strain *B. anthina* LMG 20980, identified in this study as Clade I. While strains forming Clade II be recognised as a new species, for which we propose the name *Burkholderia anthinoferrum* sp. nov. (an.thin.no.ferrum: anthino, meaning ‘from the garden’ or ‘from flowers’, referring to the origin of the majority of the isolates as previously proposed, ferrum; iron, referring to the species iron acquisition potential for siderophore synthesis). Herein, Clade I will be referred to as *B. anthina*, and Clade II will be referred to as *B. anthinoferrum*.

Since its first description in 1997, *B. anthina* has been isolated from several sources including the rhizosphere soil of gardens and house plants, commercial solutions, and from the sputum of patients with *CF* (Vandamme et al., 2002; Coenye et al., 2003). Despite the rapid expansion of *Burkholderia* genome sequences in various national databases, there are currently a paucity of genomes corresponding to *B. anthina*, with the few that do primarily originating from an environmental source. The difficulties associated with resolving the taxonomy of *Burkholderia* is particularly evident when considering the taxonomic complexity that exists with the genus. In part, this is complicated by the high level of homologous recombination between species which further blurs taxonomic boundaries (Zhou et al., 2020). In the study by Wallner et al. (2019), genomic analyses of *B. cenocepacia* revealed two distinct clades within the species that harboured differential host-specific genetic adaptations to humans and plants. Despite being closely related (>95% ANI) according to the threshold for species delimitation by ANI, it was proposed that *B. cenocepacia* be amended to recognise a new species *B. oribcola* sp. nov. From our analyses, *B. anthina* and *B. anthinoferrum* were found to be polyphyletic, having ancestrally diverged from each other, which is further reflected by differences in their genetic and functional profiles. Pan-genomic analysis demonstrated substantial genetic variance within the population. The low number of shared genes observed in the population is consistent with the pan-genome of several other *Burkholderia* species, which are considered to be highly heterogenous (Seo et al., 2015; Bochkareva et al., 2018; Zhou et al., 2020; Bach et al., 2022). Demonstrating a high evolutionary rate, it could be suggested that the necessity of constant genomic adaptation is driven by selective pressures to adapt to the environment, which corroborates the diverse environments of isolates observed in *B. anthina* (Zhou et al., 2020). In contrast, lower intra-species diversity was apparent in *B. anthinoferrum* which supports a more recent evolution in addition to phylogenetic reconstruction.

In terms of biological capabilities, *B. anthina* were significantly enriched in KEGG orthologs associated with global overview and maps (metabolic pathways, microbial metabolism in diverse environments and degradation of aromatic compounds), and xenobiotic degradation and metabolism (benzoate degradation, xylene degradation, chlorocyclohexane and chlorobenzene degradation, dioxin degradation, toluene degradation, steroid degradation, and styrene degradation). The ability to metabolise and utilise xenobiotic contaminants as a carbon or nitrogen source serves as a competitive advantage for microorganisms, particularly in nutrient-harsh environments (Miglani et al., 2022). Several bacteria including *Burkholderia, Pseudomonas, Achromobacter, Bacillus, Enterobacter* and *Alcaligenes* have remarkable bioremediation potential to degrade xenobiotic compounds from contaminated environments (Tang et al., 2016; Morya et al., 2020; Mishra et al., 2021; Miglani et al., 2022; Tirkey et al., 2022). *Burkholderia xenovorans* LB400 has been extensively studied for its ability to degrade xenobiotic compounds, including halogenated aromatics such as polychlorinated biphenyls; a highly persistent and carcinogenic pollutant resistant to environmental degradation (Erickson and Mondello, 1992; Gibson et al., 1993; Hofer et al., 1993; Furukawa, 2000; Denef et al., 2004). Similarly, *Burkholderia vietnamiensis* G4 is well studied for its potential in the degradation of trichloroethylene, a common organic ground water contaminant (Nelson et al., 1986; O'Sullivan and Mahenthiralingam, 2005).

The low bioavailability of iron and other essential metal ions in the environment is a significant nutrient limiting factor, and as such microorganisms must be equipped with robust iron acquisition mechanisms. Siderophores are small chelating compounds secreted under iron limiting conditions to sequester metal ions from the environment to increase bioavailability (Hider and Kong, 2010; Kramer et al., 2020). Siderophore-mediated iron uptake by *Burkholderia* generally consists of four types and includes ornibactin, pyochelin, cepabactin, and cepaciachelin (Thomas, 2007; Butt and Thomas, 2017). *B. anthina* is known to encode the biosynthesis of two of these iron chelating compounds, ornibactin and pyochelin, with the former siderophore displaying a peptide structure similar to that of pyoverdine produced by fluorescent pseudomonads, including *Pseudomonas aeruginosa* (Sokol et al., 2000; Agnoli et al., 2006; Butt and Thomas, 2017). In line with the literature, *B. anthina* genomes encoded the genes required for ornibactin synthesis, while *B. anthinoferrum* encoded the genes for ornibactin and pyochelin synthesis. Analysis of secondary metabolite potential revealed that *B. anthinoferrum* possessed an even wider capacity for siderophore biosynthesis than *B. anthina*, with the former encoding the biosynthetic gene clusters required for siderophore synthesis using the classical NRP pathway and the NRP-independent siderophore pathway (Challis, 2005). Specifically, an aminopolycarboxylic acid siderophore gene cluster (NRP-independent pathway) encoding the biosynthesis of EDHA was identified and exclusively present in *B. anthinoferrum* genomes. This novel iron-responsive siderophore has recently been reported in the genomes of *Streptomyces scabies*, *S. avermitilis*, *Corynebacterium pseudotuberculosis*, *C. ulcerans*, and *Nocardia brasiliensis* (Spohn et al., 2018). The identification of an additional siderophore system and several putative genes for siderophore assemble reveals an extended metabolic repertoire of *B. anthinoferrum*, which may confer an advantage in environments with insufficient iron bioavailability, such as the *CF* lung (Drevinek et al., 2008). Indeed, the production of both ornibactin and pyochelin siderophores has previously been associated with increased virulence of *Burkholderia* strains in respiratory infections and correlated with morbidity in patients with *CF* (Sokol, 1986; Sokol and Woods, 1988; Agnoli et al., 2006). From an agricultural-management perspective, siderophores also provide a microbial-based alternative for effective control against plant pathogens. Previous investigations demonstrated that *B. anthinoferrum* XXVI produced a hydroxamate siderophore with biocontrol potential against the causal agent of anthracnose, *Colletotrichum lindemuthianumn* (de Los Santos-Villalobos et al., 2012). Recent characterisation of *B. anthinoferrum* XXVI genome also identified several putative genes for siderophore synthesis, suggesting increased potential for iron acquisition via the production of chelating compounds (de Los Santos-Villalobos et al., 2018). Although several novel siderophore assembly genes were genomically identified in the genomes of *B. anthinoferrum*, it is unknown whether these siderophore systems are functional and involved in iron acquisition. Future functional studies are required to confirm that EDHA is synthesised and understand its role in scavenging iron. Taken together, our analyses show distinct differences in the genetic architecture and metabolic capabilities between *B. anthina* and *B. anthinoferrum*, and along with their polyphyletic origin, provide further evidence that these groups represent distinct species.

### Description of *Burkholderia anthinoferrum* sp. nov.

*Burkholderia anthinoferrum* sp. nov. (an.thin.no.ferrum: anthino, meaning ‘from the garden’ or ‘from flowers’, ferrum; iron). Cells are gram negative, non-motile, non-sporulating, coccobacilli bacteria. Growth is observed in LB, MacConkey and BCSA agar at 30, 37, and 42°C. Colonies in LB and BCSA are circular, convex, and cream coloured, while colonies in MacConkey are light pink coloured. Growth in BCSA produces an alkaline medium due the fermentation of lactose and sucrose and is strain dependent. The G + C content for the members of the species is 67.0%. Strains have been isolated predominately from environmental samples, but recently from clinical samples as described in this study. We propose that *Burkholderia anthinoferrum* sp. nov. XXVI be used as the type strain, a rhizosphere bacterium isolated from a mango orchid in Mexico. The strain holds a central position in the phylogeny of its clade and has been characterised in the literature (de Los Santos-Villalobos et al., 2012, 2018).

## Data availability statement

The datasets generated during this study are available on the NCBI database under the BioProject PRJNA1033429. Whole-genome sequences for Burkholderia isolates are under the Biosample accession SAMN38033600-SAMN38033617.

## Ethics statement

The studies involving humans were approved by The Prince Charles Hospital and QLD Princess Alexandra Hospital Research Ethics Committees. The studies were conducted in accordance with the local legislation and institutional requirements. The human samples used in this study were acquired from primarily isolated as part of your previous study for which ethical approval was obtained. Written informed consent for participation was not required from the participants or the participants’ legal guardians/next of kin in accordance with the national legislation and institutional requirements.

## Author contributions

AP: Conceptualization, Data curation, Investigation, Methodology, Writing – original draft, Writing – review & editing. JV: Writing – review & editing, Investigation. DC: Resources, Writing – review & editing. DS: Resources, Writing – review & editing. DR: Resources, Writing – review & editing. LB: Resources, Writing – review & editing. TW: Conceptualization, Funding acquisition, Resources, Supervision, Writing – review & editing.

## Footnotes

^1^ http://www.microbesng.com

^2^ https://www.ncbi.nlm.nih.gov/genome/

^3^ http://pubmlst.org/bcc

^4^ https://itol.embl.de/

^5^ https://github.com/geronimp/enrichM

^7^ https://github.com/antismash
