# Supplementary figures and images for: Genomic analyses of Burkholderia respiratory isolates indicates two evolutionarily distinct B. anthina clades
Source: Front Microbiol. 2023 Nov 21;14:1274280. doi: 10.3389/fmicb.2023.1274280 (PMC10702961; doi:10.3389/fmicb.2023.1274280)

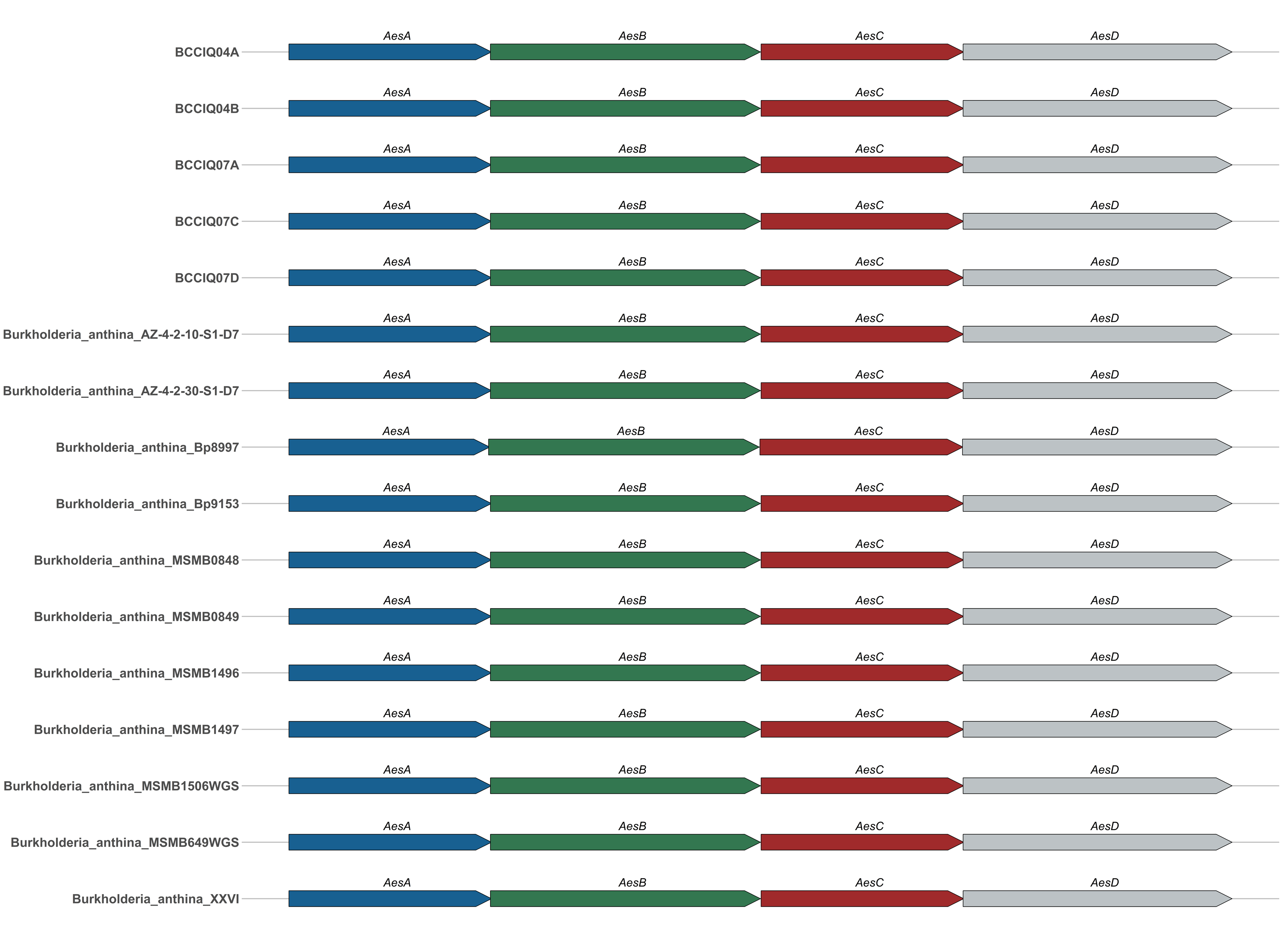

Supplement: Supplementary file 6 [file Image_1.TIF]
